# Three new Scandinavian species of *Culicoides (Culicoides)*: *Culicoides boyi* sp. nov., *Culicoides selandicus* sp. nov. and *Culicoides kalix* sp. nov. (Diptera: Ceratopogonidae)

**DOI:** 10.3897/BDJ.3.e5823

**Published:** 2015-11-26

**Authors:** Søren Achim Nielsen, Michael Kristensen, Thomas Pape

**Affiliations:** ‡ Department of Environmental, Social and Spatial Change, Roskilde University, Roskilde, Denmark; § Department of Agroecology - Entomology and Plant Pathology, Slagelse, Denmark; | Natural History Museum of Denmark, Copenhagen, Denmark

**Keywords:** Biting midges, new species, Scandinavia, morphology, molecular barcoding

## Abstract

**Background:**

In the context of a major monitoring program of *Culicoides* in Denmark and Sweden due to the appearance of bluetongue disease in 2007–2008, a large number of specimens were collected by light traps and sorted morphologically, with COI barcodes generated for selected specimens.

**New information:**

Three species are described as new to science based on both morphological and molecular data: Culicoides (Culicoides) boyi **sp. nov.** (Denmark: Jutland), C. (C.) selandicus **sp. nov.** (Denmark: Zealand) and C. (C.) kalix **sp. nov.** (Sweden: Norrbotten). All are diagnosed morphologically as well as by molecular barcoding. A key to slide-mounted females of all Scandinavian species of *Culicoides (Culicoides)* is presented.

## Introduction

Following the outbreak of bluetongue disease in 2007–2008, a major entomological monitoring program of *Culicoides* was implemented in Denmark and Sweden to collect a large material of *Culicoides* (Nielsen et al. 2010, Nielsen et al. 2014). Most specimens were sorted morphologically and some were selected for subsequent molecular barcoding in order to develop an efficient method for correct species identification (Pagès et al. 2009, Lassen et al. 2012a, Lassen et al. 2012b). One group of specimens was morphologically similar to *Culicoides pulicaris* (Linnaeus, 1758) but showed divergent cytochrome c oxidase subunit 1 (COI) barcode sequences; and two groups of specimens were morphologically similar to *C. newsteadi* (Austen, 1921) but showed divergent COI barcode sequences (Lassen et al. 2011). The three groups of specimens were denoted with informal names as *Culicoides* 'dk1', with a COI barcode diverging by 14–17% from other species of subgenus *Culicoides*, and *Culicoides* 'dk3' and *Culicoides* 'Kalix', which diverged in their COI barcodes by 5.9% from each other and by 13–18% from other species of subgenus *Culicoides* (Lassen et al. 2012b). A phylogenetic analysis clearly separated each of these three groups of specimens from all named species of *Culicoides* for which COI-barcodes were known (Lassen et al. 2012b), and based on this as well as on a detailed morphological study, the three fully diagnosable clusters of specimens were considered by Nielsen and Kristensen 2015 to represent separate species and provided with formal, scientific names. Evidence was given in Nielsen and Kristensen 2015 that a registration of the work had been done in the *Official Register of Zoological Nomenclature* (ZooBank), which after the latest amendment (International Commission on Zoological Nomenclature 2012) is required for nomenclatural acts in a digital work to be potentially available. However, the naming was not compliant with the current edition of the International Code of Zoological Nomenclature (International Commission on Zoological Nomenclature 1999) by lacking explicit fixation of name-bearing types, i.e., holotype or syntypes, for each nominal taxon (Article 16.4.1). We are here providing formal scientific names for all three species, accompanied by evidence for ZooBank registration, details of type material, and diagnostic descriptions in agreement with the current Code. Descriptions are kept to a minimum as more elaborate descriptions are found in Nielsen and Kristensen 2015. Furthermore, we provide a key to females of all Scandinavian species of *Culicoides (Culicoides)* (based on slide-mounted material).

## Materials and methods

Biting midges were collected in 2007–2008 in Sweden (Nielsen et al. 2010) and 2008–2009 in Denmark using blacklight suction traps (Lassen et al. 2012b, Nielsen et al. 2014​). The entire material of *Culicoides* was identified to species level by the first author.

For documentation of the identification, the head, wings and the posterior abdominal segments were removed from the female individuals and slide mounted, and the remaining parts of the animal were processed for DNA analysis as described by Nielsen and Kristensen 2015. All morphological measurements and molecular comparisons used in the present circumscriptions of species were carried out by Nielsen and Kristensen 2015.

The holotypes and paratypes of the three new species are deposited in the collection of the Natural History Museum of Denmark, University of Copenhagen (ZMUC).

## Taxon treatments

### Culicoides (Culicoides) boyi

Nielsen, Kristensen & Pape, 2015 sp. n.

JF766294

urn:lsid:zoobank.org:act:38841514-2435-4A35-81CA-9C9723A66A18

#### Materials

- **Type status:** Holotype. **Occurrence:** sex: Female; lifeStage: Adult; preparations: Slide (euparal); **Taxon:** order: Diptera; family: Ceratopogonidae; genus: Culicoides; subgenus: Culicoides; specificEpithet: boyi; scientificNameAuthorship: Nielsen, Kristensen & Pape, 2015; **Location:** continent: Europe; country: Denmark; countryCode: DK; county: Himmerland; municipality: Aalestrup; verbatimCoordinates: 56°40'5.13''N, 09°28'53.53''E; **Event:** samplingProtocol: UV-light suction trap; eventDate: 2008-07-09; year: 2008; month: 07; day: 09; **Record Level:** institutionCode: ZMUC
- **Type status:** Paratype. **Occurrence:** sex: Female; lifeStage: Adult; preparations: Slide (euparal); **Taxon:** order: Diptera; family: Ceratopogonidae; genus: Culicoides; subgenus: Culicoides; specificEpithet: boyi; scientificNameAuthorship: Nielsen, Kristensen & Pape, 2015; **Location:** continent: Europe; country: Denmark; countryCode: DK; county: Himmerland; municipality: Aalestrup; verbatimCoordinates: 56°40'5.13''N, 09°28'53.53''E; **Event:** samplingProtocol: UV-light suction trap; eventDate: 2008-07-09; year: 2008; month: 07; day: 09; **Record Level:** institutionCode: ZMUC
- **Type status:** Paratype. **Occurrence:** sex: Female; lifeStage: Adult; preparations: Slide (euparal); **Taxon:** order: Diptera; family: Ceratopogonidae; genus: Culicoides; subgenus: Culicoides; specificEpithet: boyi; scientificNameAuthorship: Nielsen, Kristensen & Pape, 2015; **Location:** continent: Europe; country: Denmark; countryCode: DK; county: Himmerland; municipality: Aalestrup; verbatimCoordinates: 56°40'5.13''N, 09°28'53.53''E; **Event:** samplingProtocol: UV-light suction trap; eventDate: 2008-07-09; year: 2008; month: 07; day: 09; **Record Level:** institutionCode: ZMUC
- **Type status:** Paratype. **Occurrence:** sex: Female; lifeStage: Adult; preparations: Slide (euparal); **Taxon:** order: Diptera; family: Ceratopogonidae; genus: Culicoides; subgenus: Culicoides; specificEpithet: boyi; scientificNameAuthorship: Nielsen, Kristensen & Pape, 2015; **Location:** continent: Europe; country: Denmark; countryCode: DK; county: Himmerland; municipality: Aalestrup; verbatimCoordinates: 56°40'5.13''N, 09°28'53.53''E; **Event:** samplingProtocol: UV-light suction trap; eventDate: 2008-07-09; year: 2008; month: 07; day: 09; **Record Level:** institutionCode: ZMUC
- **Type status:** Paratype. **Occurrence:** sex: Female; lifeStage: Adult; preparations: Slide (euparal); **Taxon:** order: Diptera; family: Ceratopogonidae; genus: Culicoides; subgenus: Culicoides; specificEpithet: boyi; scientificNameAuthorship: Nielsen, Kristensen & Pape, 2015; **Location:** continent: Europe; country: Denmark; countryCode: DK; county: Himmerland; municipality: Aalestrup; verbatimCoordinates: 56°40'5.13''N, 09°28'53.53''E; **Event:** samplingProtocol: UV-light suction trap; eventDate: 2008-07-09; year: 2008; month: 07; day: 09; **Record Level:** institutionCode: ZMUC
- **Type status:** Paratype. **Occurrence:** sex: Female; lifeStage: Adult; preparations: Slide (euparal); **Taxon:** order: Diptera; family: Ceratopogonidae; genus: Culicoides; subgenus: Culicoides; specificEpithet: boyi; scientificNameAuthorship: Nielsen, Kristensen & Pape, 2015; **Location:** continent: Europe; country: Denmark; countryCode: DK; county: Himmerland; municipality: Aalestrup; verbatimCoordinates: 56°40'5.13''N, 09°28'53.53''E; **Event:** samplingProtocol: UV-light suction trap; eventDate: 2009-07-22; year: 2009; month: 07; day: 22; **Record Level:** institutionCode: ZMUC
- **Type status:** Paratype. **Occurrence:** sex: Female; lifeStage: Adult; preparations: Slide (euparal); **Taxon:** order: Diptera; family: Ceratopogonidae; genus: Culicoides; subgenus: Culicoides; specificEpithet: boyi; scientificNameAuthorship: Nielsen, Kristensen & Pape, 2015; **Location:** continent: Europe; country: Denmark; countryCode: DK; county: Himmerland; municipality: Aalestrup; verbatimCoordinates: 56°40'5.13''N, 09°28'53.53''E; **Event:** samplingProtocol: UV-light suction trap; eventDate: 2009-07-22; year: 2009; month: 07; day: 22; **Record Level:** institutionCode: ZMUC
- **Type status:** Paratype. **Occurrence:** sex: Female; lifeStage: Adult; preparations: Slide (euparal); **Taxon:** order: Diptera; family: Ceratopogonidae; genus: Culicoides; subgenus: Culicoides; specificEpithet: boyi; scientificNameAuthorship: Nielsen, Kristensen & Pape, 2015; **Location:** continent: Europe; country: Denmark; countryCode: DK; county: Himmerland; municipality: Nibe; verbatimCoordinates: 56°54'21.05''N, 09°37'23.90''E; **Event:** samplingProtocol: UV-light suction trap; eventDate: 2008-07-09; year: 2009; month: 07; day: 09; **Record Level:** institutionCode: ZMUC
- **Type status:** Paratype. **Occurrence:** sex: Female; lifeStage: Adult; preparations: Slide (euparal); **Taxon:** order: Diptera; family: Ceratopogonidae; genus: Culicoides; subgenus: Culicoides; specificEpithet: boyi; scientificNameAuthorship: Nielsen, Kristensen & Pape, 2015; **Location:** continent: Europe; country: Denmark; countryCode: DK; county: Mors; municipality: Nykøbing Mors; verbatimCoordinates: 56°53'55.39''N, 08°48'41.65''E; **Event:** samplingProtocol: UV-light suction trap; eventDate: 2008-07-09; year: 2009; month: 07; day: 09; **Record Level:** institutionCode: ZMUC

#### Description

Female: Eyes bare and contiguous dorsally (Fig. 1a), length of contact divided by diameter of one ocellus = 1.5 ± 0.2. Length of antennal flagellum 746 ± 44 µm. Antennal ratio (AR: length of flagellomeres 9–13 divided by length of flagellomeres 1–8) 1.03 ± 0.04, number of sensilla coeloconica 14.71 ± 1.26. First flagellomere with 3.65 ± 0.24 sensilla coeloconica, long and slender (Fig. 1b). First flagellomere ratio (length divided by width) = 1.78 ± 0.07 (Fig. 1b​). Maxillary palp ratio (PR: length/width of third palpal segment) = 2.9 ± 0.3 (Fig. 1c), P3/P2 (length of third palp segment divided by length of second) = 1.04 ± 0.11 (Fig. 1c). Numbers of mandibular and maxillary teeth = 15.1 ± 1.2 and 17.1 ± 1.4, respectively. Ratio of mandibular versus maxillar teeth (M/M) = 1.13 ± 0.10. Head height/proboscis length ratio (H/P) = 1.29 ± 0.07.

Wing length 1,641 ± 10 μm (Fig. 1d, e​). The dark hourglass-shaped mark in the centre of cell r_3_ is broadest above the longitudinal fold above M_1_. Wing markings light brownish, distinct from the remaining, hyaline wing membrane. Cubital cell usually with a distinct spot (Fig. 1d, e; observed in about two thirds of the specimens), but this spot may be very small or entirely absent.

Two normal-sized spermathecae, ovoid, of almost equal size, with a short neck and a third rudimentary one (Fig. 1f​).

Male: Unknown.

#### Etymology

Named as a tribute to Boy Overgaard Nielsen, an outstanding Danish entomologist and current emeritus at Aarhus University.

#### Distribution

**Palaearctic** – Denmark (northern Jutland).

#### Taxon discussion

The length divided by the width of the first flagellomere is significantly higher in *C. boyi* compared to all other Scandinavian species of this subgenus.

### Culicoides (Culicoides) selandicus

Nielsen, Kristensen & Pape, 2015 sp. n.

JF766324

urn:lsid:zoobank.org:act:9F4C2671-824C-4E92-B954-3385EF769DC7

#### Materials

- **Type status:** Holotype. **Occurrence:** sex: Female; lifeStage: Adult; preparations: Slide (euparal); **Taxon:** order: Diptera; family: Ceratopogonidae; genus: Culicoides; subgenus: Culicoides; specificEpithet: selandicus; scientificNameAuthorship: Nielsen, Kristensen & Pape, 2015; **Location:** continent: Europe; country: Denmark; countryCode: DK; county: Næstved; verbatimCoordinates: 55°11'20.15''N, 11°47'58.61''E; **Event:** samplingProtocol: UV-light suction trap; eventDate: 2008-07-08; year: 2008; month: 07; day: 08; **Record Level:** institutionCode: ZMUC
- **Type status:** Paratype. **Occurrence:** sex: Female; lifeStage: Adult; preparations: Slide (euparal); **Taxon:** order: Diptera; family: Ceratopogonidae; genus: Culicoides; subgenus: Culicoides; specificEpithet: selandicus; scientificNameAuthorship: Nielsen, Kristensen & Pape, 2015; **Location:** continent: Europe; country: Denmark; countryCode: DK; county: Næstved; verbatimCoordinates: 55°11'20.15''N, 11°47'58.61''E; **Event:** samplingProtocol: UV-light suction trap; eventDate: 2008-07-08; year: 2008; month: 07; day: 08; **Record Level:** institutionCode: ZMUC
- **Type status:** Paratype. **Occurrence:** sex: Female; lifeStage: Adult; preparations: Slide (euparal); **Taxon:** order: Diptera; family: Ceratopogonidae; genus: Culicoides; subgenus: Culicoides; specificEpithet: selandicus; scientificNameAuthorship: Nielsen, Kristensen & Pape, 2015; **Location:** continent: Europe; country: Denmark; countryCode: DK; county: Næstved; verbatimCoordinates: 55°11'20.15''N, 11°47'58.61''E; **Event:** samplingProtocol: UV-light suction trap; eventDate: 2008-07-08; year: 2008; month: 07; day: 08; **Record Level:** institutionCode: ZMUC
- **Type status:** Paratype. **Occurrence:** sex: Female; lifeStage: Adult; preparations: Slide (euparal); **Taxon:** order: Diptera; family: Ceratopogonidae; genus: Culicoides; subgenus: Culicoides; specificEpithet: selandicus; scientificNameAuthorship: Nielsen, Kristensen & Pape, 2015; **Location:** continent: Europe; country: Denmark; countryCode: DK; county: Næstved; verbatimCoordinates: 55°11'20.15''N, 11°47'58.61''E; **Event:** samplingProtocol: UV-light suction trap; eventDate: 2008-07-08; year: 2008; month: 07; day: 08; **Record Level:** institutionCode: ZMUC
- **Type status:** Paratype. **Occurrence:** sex: Female; lifeStage: Adult; preparations: Slide (euparal); **Taxon:** order: Diptera; family: Ceratopogonidae; genus: Culicoides; subgenus: Culicoides; specificEpithet: selandicus; scientificNameAuthorship: Nielsen, Kristensen & Pape, 2015; **Location:** continent: Europe; country: Denmark; countryCode: DK; county: Næstved; verbatimCoordinates: 55°11'20.15''N, 11°47'58.61''E; **Event:** samplingProtocol: UV-light suction trap; eventDate: 2008-07-08; year: 2008; month: 07; day: 08; **Record Level:** institutionCode: ZMUC

#### Description

Female: Eyes bare and contiguous dorsally (Fig. 2a), length of contact divided by diameter of one ocellus = 1.5 ± 0.8. Length of antennal flagellum 616.3 ± 10 μm, antennal ratio (AR: length of flagellomeres 9–13 divided by length of flagellomeres 1–8) = 1.12 ± 0.04, number of sensilla coeloconica = 12.29 ± 0.95. First flagellomere with 3.65 ± 0.70 sensilla coeloconica (Fig. 2b). First flagellomere ratio (length divided by width) = 1.56 ± 0.11 (Fig. 2b). Maxillary palp ratio (PR: length/width of third palpal segment) = 3.2 ± 0.3 (Fig. 2c), P3/P2 (length of third palpal segment divided by length of second) = 0.96 ± 0.06 (Fig. 2c). Numbers of mandibular and maxillary teeth = 15.0 ± 1.0 and 19.6 ± 1.5, respectively. Ratio of mandibular vs. maxillary teeth (M/M) = 1.31 ± 0.12. Head height/proboscis length ratio (H/P) = 1.16 ± 0.06.

Wing length 1,339 ± 33 μm (Fig. 2d, e). The dark hourglass-shaped mark in the centre of cell r_3_ is broadest above the longitudinal fold above M_1_. The dark areas on the wing are extensive and encompassing vein M_1_ and sometimes M_2_. Small pale spots may be found at the tip of veins M_1_ and M_2_. Wing with a large dark spot in cell cu separated from the dark areas bordering Cu_1_ and Cu_2_ (Fig. 2d, e​).

Spermathecae not observed.

Male: Unknown.

#### Etymology

The species epithet refers to the name of the major Danish island Sjælland (Latin = Selandia; English = "Zealand" or more rarely "Sealand"), where the type series was collected.

#### Distribution

**Palaearctic** – Denmark (Zealand).

#### Taxon discussion

*Culicoides selandicus* may be confused with *C. kalix* but differs habitually by the extensive dark areas on the wings. The head/proboscis ratio of *C. selandicus* (1.16 ± 0.06) is smaller than in *C. kalix* (1.29 ± 0.07), although a small overlap should be expected when more specimens are measured. The average number of antennal sensilla coeloconica (12.29±0.95) is higher than in *C. kalix* (10.70 ± 0.82), and the first flagellomere has a higher average of sensilla (4.1) compared to *C. kalix* (3.0). The P3/P2 ratio (0.96 ± 0.06) differs from that of *C. kalix* (0.87 ± 0.08), but with a large overlap. Second maxillary palp segment is about as long as the third, while in *C. kalix* the second palp segment is longer than the third. Third maxillary palp segment is more slender (PR = 3.2 ± 0.3) than that of *C. kalix* (PR = 2.9 ± 0.2). Maxillary palp segments four and five of equal length provides a difference from *C. kalix*, where the fifth palp segment is longer than the fourth. The ratio of mandibular vs. maxillary teeth is significantly higher in *C. selandicus* (1.31 ± 0.12) than in *C. kalix* (1.17 ± 0.12).

### Culicoides (Culicoides) kalix

Nielsen, Kristensen & Pape, 2015 sp. n.

JF766329

urn:lsid:zoobank.org:act:A1B4ADB9-C7C7-4FE1-9607-D126713394E9

#### Materials

- **Type status:** Holotype. **Occurrence:** sex: Female; lifeStage: Adult; preparations: Slide (euparal); **Taxon:** order: Diptera; family: Ceratopogonidae; genus: Culicoides; subgenus: Culicoides; specificEpithet: kalix; scientificNameAuthorship: Nielsen, Kristensen & Pape, 2015; **Location:** continent: Europe; country: Sweden; countryCode: SE; county: Norrbotten; municipality: Kalix; verbatimCoordinates: 65°44'45.13''N, 23°03'55.62''E; **Event:** samplingProtocol: UV-light suction trap; eventDate: 2008-08-23; year: 2008; month: 08; day: 23; **Record Level:** institutionCode: ZMUC
- **Type status:** Paratype. **Occurrence:** sex: Female; lifeStage: Adult; preparations: Slide (euparal); **Taxon:** order: Diptera; family: Ceratopogonidae; genus: Culicoides; subgenus: Culicoides; specificEpithet: kalix; scientificNameAuthorship: Nielsen, Kristensen & Pape, 2015; **Location:** continent: Europe; country: Sweden; countryCode: SE; county: Norrbotten; municipality: Kalix; verbatimCoordinates: 65°44'45.13''N, 23°03'55.62''E; **Event:** samplingProtocol: UV-light suction trap; eventDate: 2008-08-23; year: 2008; month: 08; day: 23; **Record Level:** institutionCode: ZMUC
- **Type status:** Paratype. **Occurrence:** sex: Female; lifeStage: Adult; preparations: Slide (euparal); **Taxon:** order: Diptera; family: Ceratopogonidae; genus: Culicoides; subgenus: Culicoides; specificEpithet: kalix; scientificNameAuthorship: Nielsen, Kristensen & Pape, 2015; **Location:** continent: Europe; country: Sweden; countryCode: SE; county: Norrbotten; municipality: Kalix; verbatimCoordinates: 65°44'45.13''N, 23°03'55.62''E; **Event:** samplingProtocol: UV-light suction trap; eventDate: 2008-08-23; year: 2008; month: 08; day: 23; **Record Level:** institutionCode: ZMUC
- **Type status:** Paratype. **Occurrence:** sex: Female; lifeStage: Adult; preparations: Slide (euparal); **Taxon:** order: Diptera; family: Ceratopogonidae; genus: Culicoides; subgenus: Culicoides; specificEpithet: kalix; scientificNameAuthorship: Nielsen, Kristensen & Pape, 2015; **Location:** continent: Europe; country: Sweden; countryCode: SE; county: Norrbotten; municipality: Kalix; verbatimCoordinates: 65°44'45.13''N, 23°03'55.62''E; **Event:** samplingProtocol: UV-light suction trap; eventDate: 2008-08-23; year: 2008; month: 08; day: 23; **Record Level:** institutionCode: ZMUC
- **Type status:** Paratype. **Occurrence:** sex: Female; lifeStage: Adult; preparations: Slide (euparal); **Taxon:** order: Diptera; family: Ceratopogonidae; genus: Culicoides; subgenus: Culicoides; specificEpithet: kalix; scientificNameAuthorship: Nielsen, Kristensen & Pape, 2015; **Location:** continent: Europe; country: Sweden; countryCode: SE; county: Norrbotten; municipality: Kalix; verbatimCoordinates: 65°44'45.13''N, 23°03'55.62''E; **Event:** samplingProtocol: UV-light suction trap; eventDate: 2008-08-23; year: 2008; month: 08; day: 23; **Record Level:** institutionCode: ZMUC

#### Description

Female: Eyes bare and contiguous dorsally (Fig. 3a​), length of contact contact divided by diameter of one ocellus = 1.2 ± 0.3. Length of antennal flagellum 646 ± 14 μm. Antennal ratio (AR: length of flagellomeres 9–13 divided by length of flagellomeres 1–8) 1.13 ± 0.04, number of sensilla coeloconica 10.70 ± 0.82. First flagellomere with 3.0 ± 0.0 sensilla coeloconica. First flagellomere ratio (length divided by width) = 1.46 ± 0.06. Maxillary palp ratio (PR: length/width of third palp segment) = 2.9 ± 0.2 (Fig. 3c), P3/P2 (length of third palp segment divided by length of second) = 0.87 ± 0.08 (Fig. 3c). Numbers of mandibular and maxillary teeth = 12.8 ± 0.6 and 14.9 ± 1.5, respectively. Ratio of mandibular vs. maxillary teeth (M/M) = 1.17 ± 0.12. Head height/proboscis length ratio (H/P) = 1.29 ± 0.07.

Wing length 1,423 ± 39 μm (Fig. 3d, e). The shape of the dark hour-glass formed mark in the middle of r_3_ is broadest above the longitudinal fold above M_1_. The dark areas on the wings are extensive and surrounding vein M_1_ and M_2_. Wings have a large dark spot in cell cu, which is separated from the dark areas bordering Cu_1_ and Cu_2_ (Fig. 3d, e).

Two functional spermathecae, ovoid, of almost equal size, with a short neck; a third rudimentary one present (Fig. 3f).

Male: Unknown.

#### Etymology

The species epithet refers to the municipality Kalix in northern Sweden, in which the type series was collected.

#### Distribution

**Palaearctic** – ​Sweden (Norrbotten).

#### Taxon discussion

*Culicoides kalix* could be confused with *C. selandicus* but may be separated from this as already discussed above under the description of the latter species and as outlined in the key.

## Identification Keys

### Key to females of Scandinavian species of *Culicoides (Culicoides)*

**Table:** 

1	Wing with a spot in cell cu	2
–	Wing without a spot in cell cu	9
2	Third segment of maxillary palp about as long as or longer than the second segment (P3/P2 ≥ 0.94). Wing with the dark hour-glass mark in cell r_3_ broadest above the longitudinal fold above vein M_1_	3
–	Third segment of maxillary palp shorter than the second segment (P3/P2 ≤ 0.93). Wing with the dark hour-glass mark in cell r_3_ broadest above the longitudinal fold above vein M_1_ or broadest at or below the fold above M_1_	6
3	Wing darkened around the entire length of vein M_1_	4
–	Wing darkened for some part of vein M_1_	5
4	Wing with two dark marks in cell m_1_. The dark hour-glass mark in cell r_3_ is broad and roughly square in outline. Third segment of maxillary palp longer than second segment (P3/P2 > 1.01); PR (palp ratio, i.e., length/width of third segment) < 2.8. Fronto-vertex/ocellus ratio < 0.78	***C. newsteadi* Austen** & ***C. halophilus*Kieffer** (separation based on morphological characters currently not possible)
–	Wing with only one dark mark in cell m_1_. The dark hour-glass mark in cell r_3_ is not broad and square in outline. Third segment of maxillary palp at most as long as second segment (P3/P2 ≤ 1.0); PR > 3.0. Fronto-vertex/ocellus ratio > 0.87	***C. selandicus* Nielsen, Kristensen & Pape, sp. nov.**
5	Wing with small pale spots at the tip of veins M_1_, M_2_, and Cu_1_. AR (antennal ratio, i.e., length of flagellomeres 9–13 divided by the length of flagellomeres 1–8) > 1.08. Ratio length/width of first flagellomere < 1.6	***C. punctatus* (Meigen)**
–	Wing without spots at the tip of veins M_1_, M_2_ and Cu_1_. AR < 1.08. Ratio length/width of first flagellomere > 1.7	***C. boyi* Nielsen, Kristensen & Pape, sp. nov. (part)**
6	Wing with the dark hour-glass mark in cell r_3_ broadest above the longitudinal fold above M_1_ or hour-glass mark with continuous outline and of equal width above and at the longitudinal fold above vein M_1_	7
–	Wing with the dark hour-glass mark in cell r_3_ broadest at or below the longitudinal fold above vein M_1_	8
7	Wing with the dark hour-glass mark in r_3_ broadest above the longitudinal fold above vein M_1_. The dark areas in wings surround the entire length of the veins M_1_ and M_2_. Mandibular teeth 12–14, maxillary teeth 12–17. Number of antennal sensilla coeloconica 9–12	***C. kalix* Nielsen, Kristensen & Pape, sp. nov.**
–	Wing with the dark hour-glass mark in cell r_3_ with continuous outline and equal widths above the longitudinal fold and at the fold above vein M_1_. The dark areas in wings do not surround veins M_1_ and M_2_. Mandibular teeth 16–20, maxillary teeth 19–21. Number of antennal sensilla coeloconica 12–19	***C. pulicaris* (Linnaeus)**
8	Small species (wing length < 1400 µm). Wing markings are faint but sharply defined. Third segment of maxillary palp rhomboid. AR (antennal ratio, i.e., length of flagellomeres 9–13 divided by the length of flagellomeres 1–8) < 1.09. Head/proboscis ratio > 1.26. Number of antennal sensilla coeloconica 7–12	***C. impunctatus* Goetghebuer (part)**
–	Large species (wing length > 1600 µm). Wing with extensive and vaguely defined dark markings. Third segment of maxillary palp cigar-shaped. AR > 1.09. Head/proboscis ratio < 1.24. Number of antennal sensilla coeloconica 12–20	***C. deltus* Downes and Kettle (part)**
9	Wing with distinct dark markings; the dark hour-glass mark in the middle of cell r_3_ broadest above the longitudinal fold above the longitudinal fold above cell M_1_	***C. boyi* Nielsen, Kristensen & Pape, sp. nov. (part)**
–	Wing with vaguely defined markings; the dark hour-glass mark in cell r_3_ broadest at or below the fold above M_1_	10
10	PR (palp ratio, i.e., length/width of third segment) < 3.5. Fronto-vertex/ocellus ratio > 1.2	11
–	PR > 3.5. Fronto-vertex/ocellus ratio < 1.0	***C. grisescens* Edwards**
11	Small species (wing length < 1400 µm). Third segment of maxillary palp rhomboid. AR (antennal ratio, i.e., length of flagellomeres 9–13 divided by the length of flagellomeres 1–8) < 1.09. Head/proboscis ratio > 1.26. Number of antennal sensilla coeloconica 7–12	***C. impunctatus* Goetghebuer (part)**
–	Large species (wing length > 1600 µm). Third segment of maxillary palp cigar-shaped. AR > 1.09. Head/proboscis ratio < 1.24. Number of antennal sensilla coeloconica 12–20	***C. deltus* Edwards (part)**

The problems in constructing reliable keys to adults of the European species of *Culicoides (Culicoides)* due to overlapping morphometric measures are well known (e.g., Campbell and Pelham-Clinton 1960). Lane 1981 showed how a combination of wing pattern elements and (other) quantitative characters would increase the taxonomic resolution, but we acknowledge that the present key may not be able to allocate every adult individual unambiguously to its 'true' taxonomic species.

Note that we are following Borkent 2014 in treating *C. deltus* and *C. lupicaris* as synonyms. Lassen et al. 2012a treated *C. halophilus* as a species distinct from *C. newsteadi* based on molecular data, but as we have been unable to find morphological features separating these taxa, they will key out together.

## Analysis

Relevant comparisons for separating the three new species from their morphologically most similar Scandinavian congeners are given in Table 1​ based on data provided by Nielsen and Kristensen 2015.

## Discussion

The recent arrival of bluetongue virus in northern and western Europe (Wilson and Mellor 2009) brought an increased interest in re-evaluating the capacity and importance of European/Palaearctic *Culicoides* species in transmission, which again put increased focus on delimiting and identifying the species found in Europe (Carpenter et al. 2009). Several studies have pointed to the existence of cryptic species, which were indicated primarily by molecular 'barcoding' techniques (e.g., Pagès et al. 2009, Ander et al. 2012, Lassen et al. 2012b, Augot et al. 2013, Sarvašová et al. 2014), and a growing amount of morphological data has brought support to their validity (e.g., Augot et al. 2013, Nielsen and Kristensen 2015). Molecular and morphological data have been shown to be widely congruent for northern European species (Stur and Borkent 2014), and where highly divergent barcode clusters are found within morphological species, this has usually been interpreted as potentially cryptic species (Ander et al. 2012, Lassen et al. 2012b, Wenk et al. 2012, Stur and Borkent 2014).

The European fauna of *Culicoides (Culicoides)* is in need of a thorough taxonomic revision based on both morphological and molecular data, but this will be a massive undertaking that reaches far beyond the scope of the present paper. The Scandinavian fauna of *Culicoides (Culicoides)* has been sampled extensively as a result of the recent bluetongue epidemic, and the current study is based on thousands of specimens examined [by SAN] from many localities widely distributed through Denmark and Sweden. The three species here described as new do not match any of the European species as keyed by Mathieu et al. 2012 nor any of the species in the key provided by Glukhova 2005 for the Russian fauna.

Revisionary taxonomy of European Ceratopogonidae suffers from the well-known constraints of old names and insufficient or missing type material. A number of nominal species currently listed in synonymy under *C. newsteadi* and *C. pulicaris* (see Borkent 2014) could in principle provide the valid name for one or more of the taxa described in the present paper. However, no types appear to exist for the nominal species of *Culicoides* described by Jean-Jacques Kieffer (B. Mathieu, personal communication June 2015), which represent the majority of these synonyms, and neotypifications of these old nominal species would seem to have little justification without a much more complete European sampling than what has been available to us.

**Figure 4.**
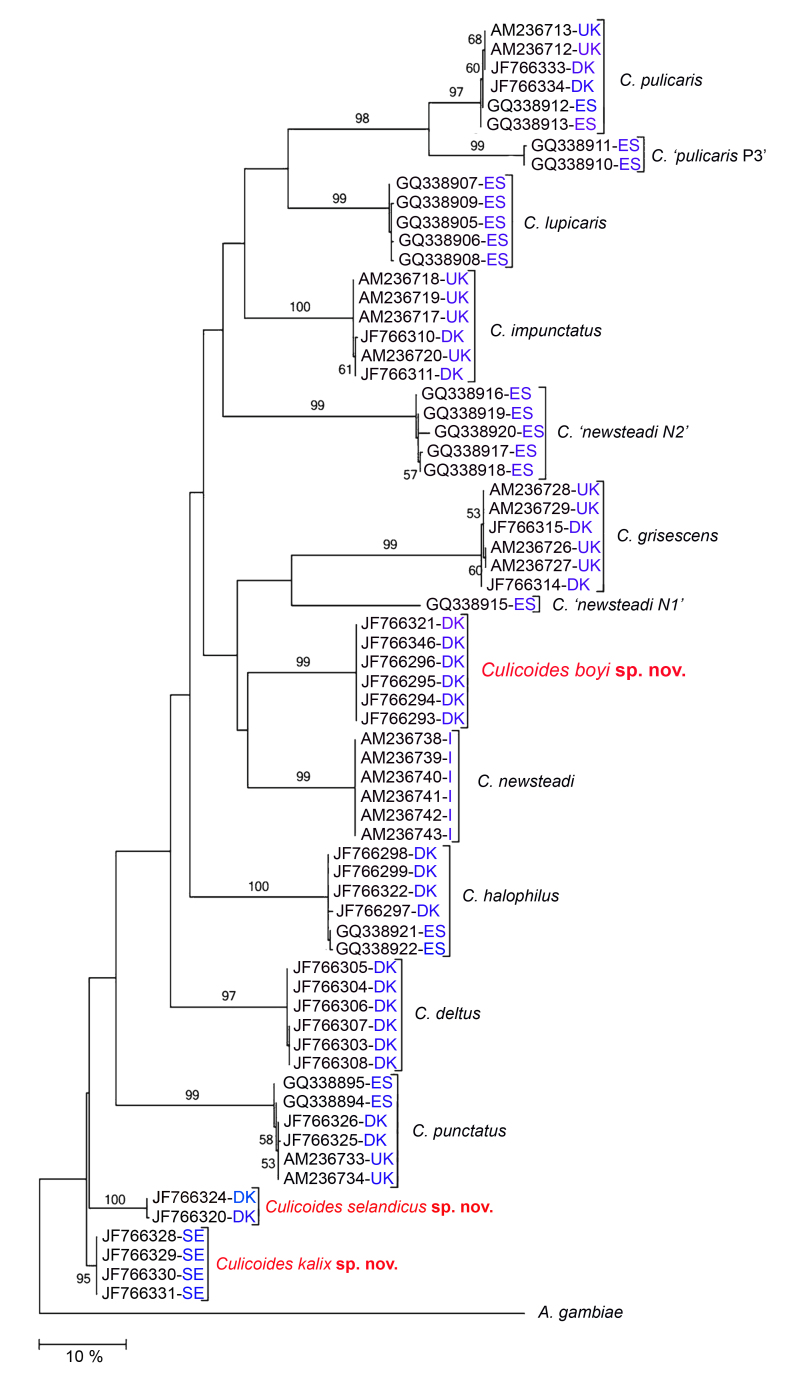
Cladogram based on COI-data showing clusters of species of *Culicoides* (*s.str.*) from western Europe. GenBank numbers with a concluding country code to show geographic origin. Numbers on branches are bootstrap values, and branch lengths are equivalent to computed evolutionary distance (scale at bottom). Redrawn from Lassen et al. 2012b, to whom is referred for details of the analysis.

The molecular data from the three species named in the present paper were analysed by Lassen et al. 2012b in a study incorporating barcode data from specimens across Europe. Their resulting cladogram is here redrawn and shown with clear indication of the country of origin for each specimen (Fig. 4). All three species described in the present paper show an evolutionary distance to their nearest neighbour much above the 3% suggested by Hebert et al. 2003 to indicate specific separation.

It is noteworthy that the three new species were collected at single locations or from a few locations in close proximity in spite of a very large sampling. These species are most likely more widely distributed, as are the majority of the well-known biting midge species, and what may look like a restricted geographical occurrence may be due to either a patchy distribution or a very short adult flying period.

## Supplementary Material

XML Treatment for Culicoides (Culicoides) boyi

XML Treatment for Culicoides (Culicoides) selandicus

XML Treatment for Culicoides (Culicoides) kalix

## Figures and Tables

**Figure 1a.**
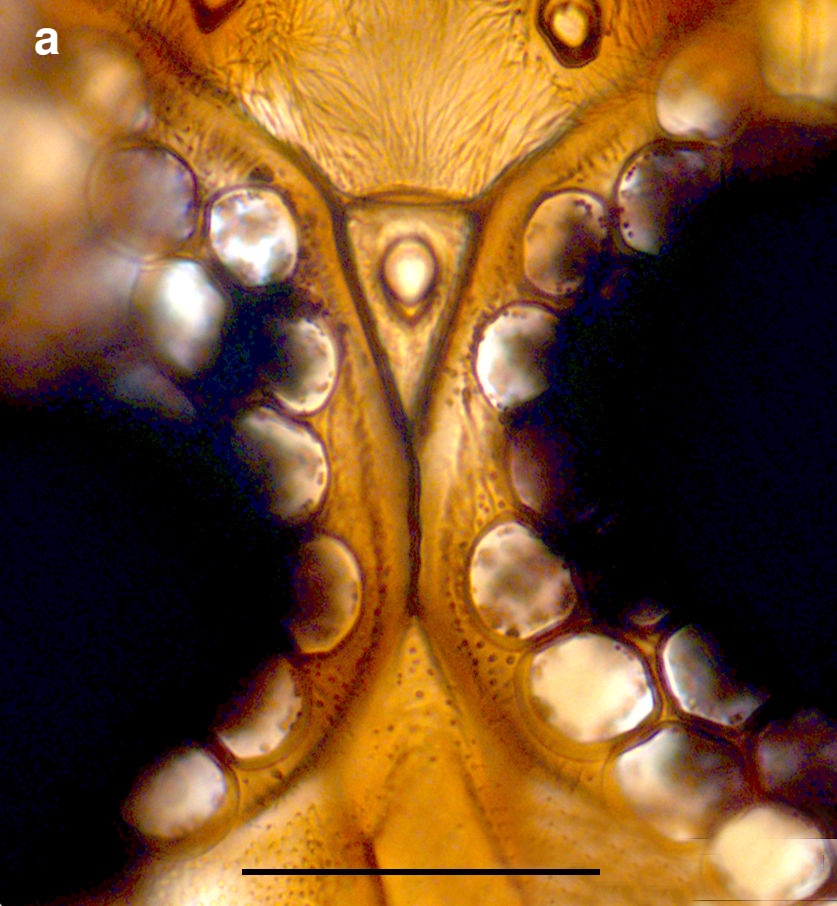
Vertex. Scale = 50 µm.

**Figure 1b.**
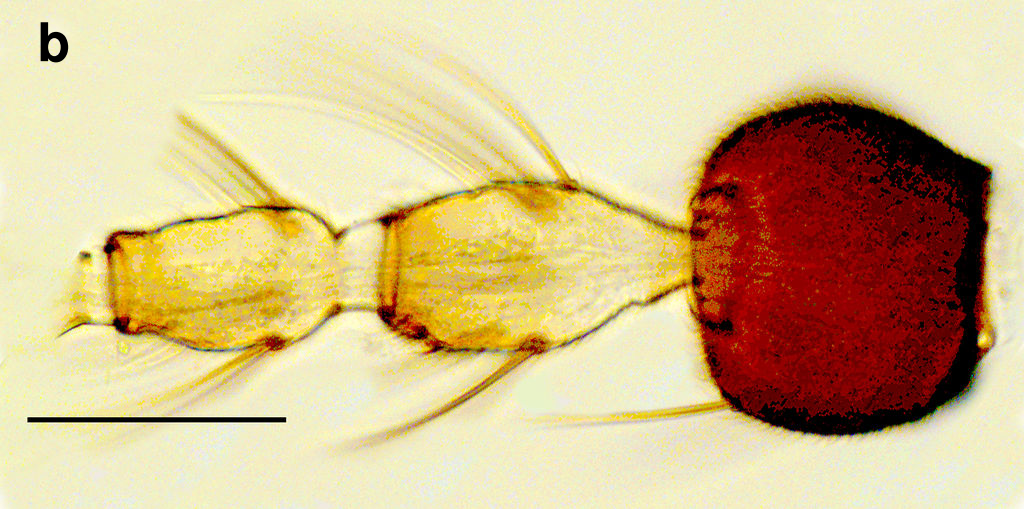
Antennal pedicellus and first two flagellomeres. Scale = 50 µm.

**Figure 1c.**
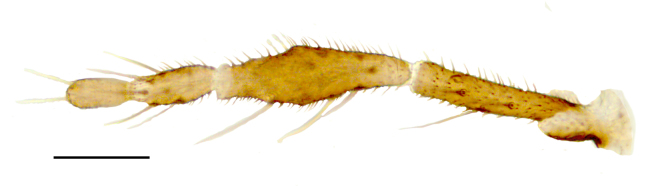
Left palp, dorsal view. Scale = 50 µm.

**Figure 1d.**
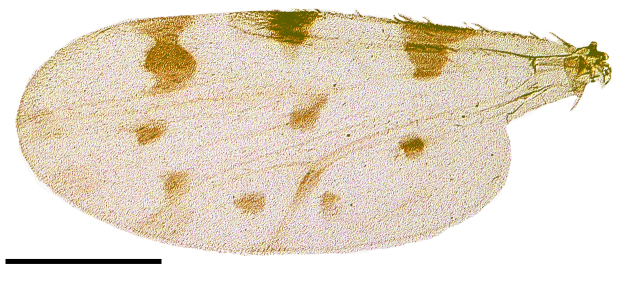
Wing, brightfield photo. Scale = 500 µm.

**Figure 1e.**
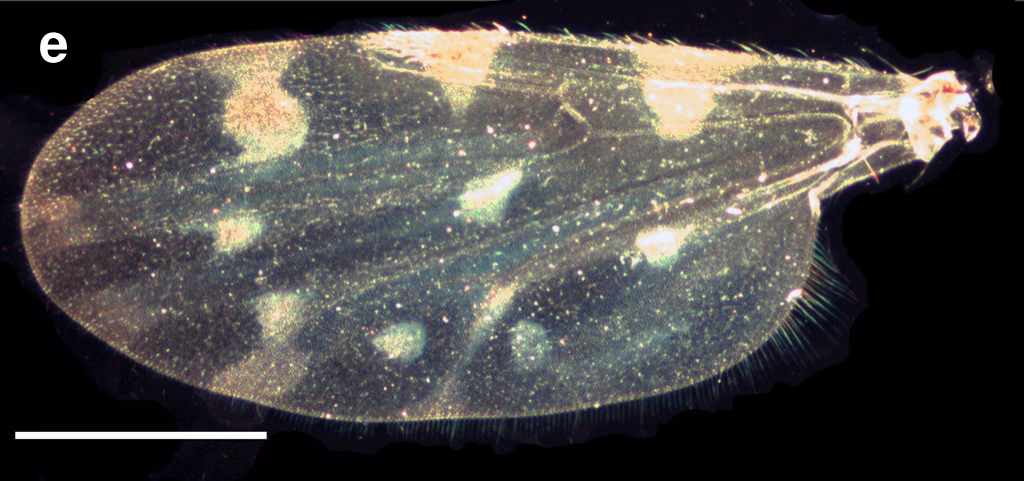
Wing, darkfield photo. Scale = 500 µm.

**Figure 1f.**
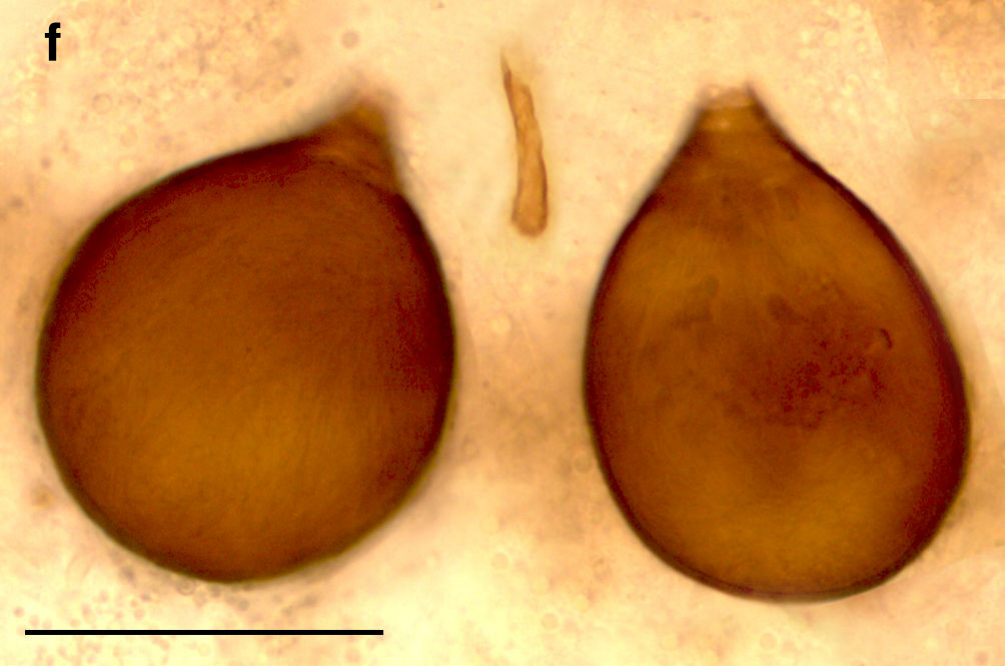
Spermathecae. Scale = 50 µm

**Figure 2a.**
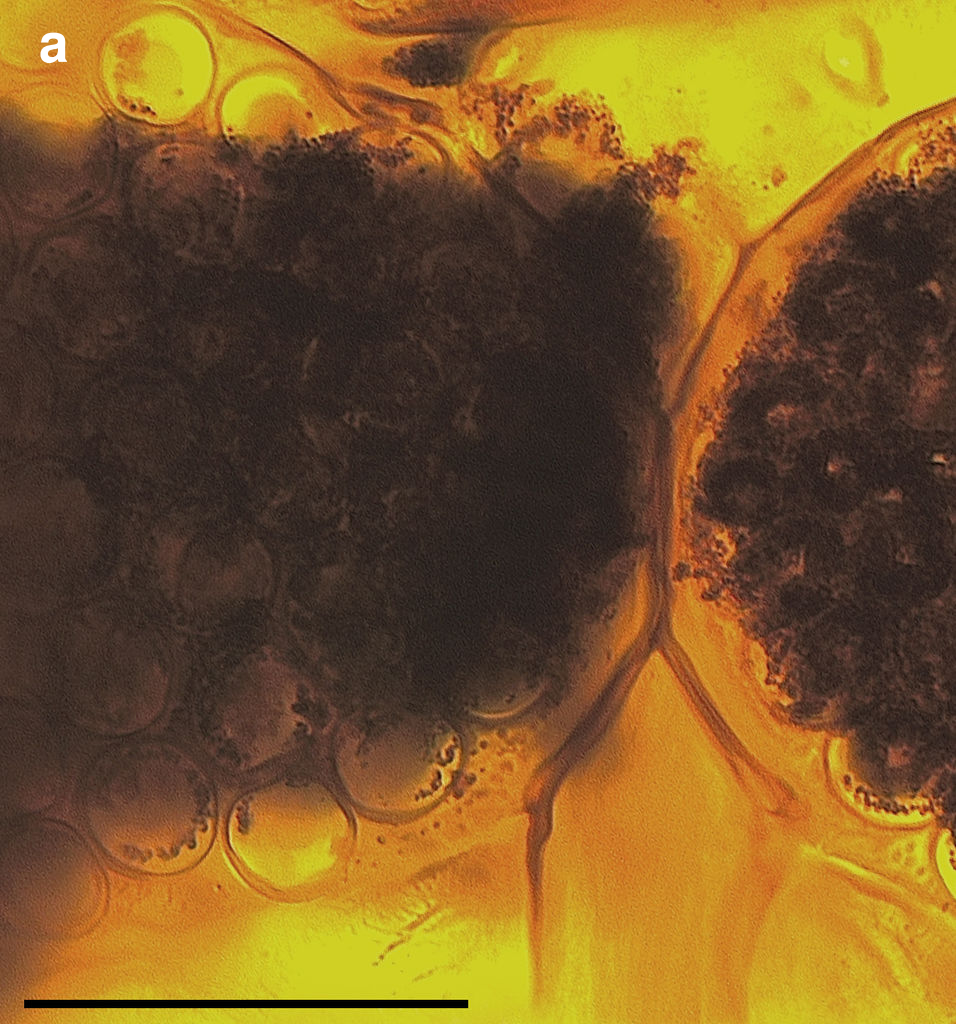
Vertex. Scale = 50 µm.

**Figure 2b.**
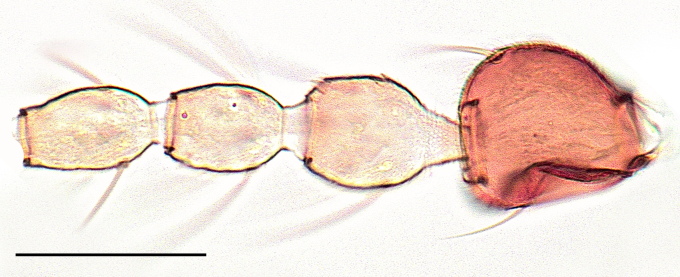
Antennal pedicellus and first three flagellomeres. Scale = 50 µm.

**Figure 2c.**
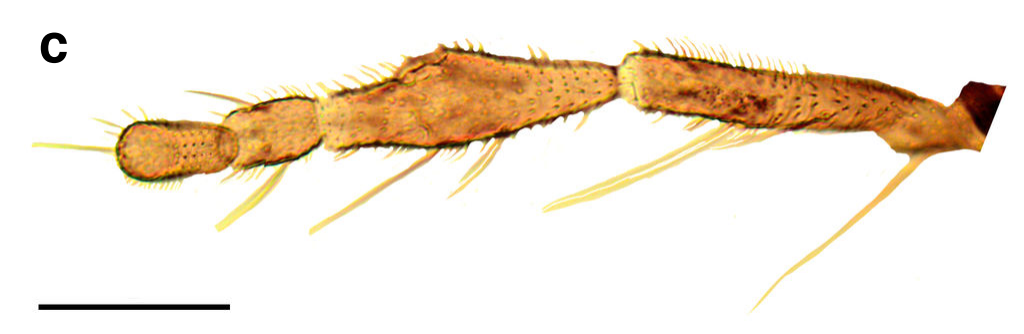
Left palp, dorsal view. Scale = 50 µm.

**Figure 2d.**
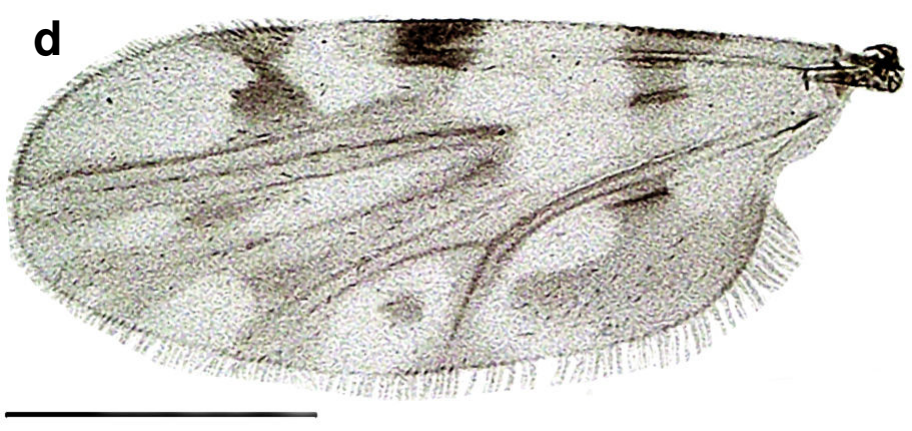
Wing, brightfield photo. Scale = 500 µm.

**Figure 2e.**
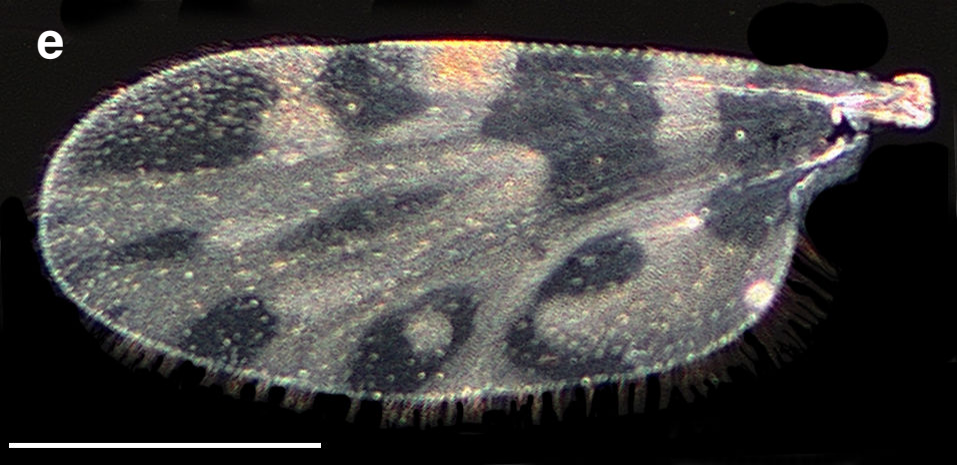
Wing, darkfield photo. Scale = 500 µm.

**Figure 3a.**
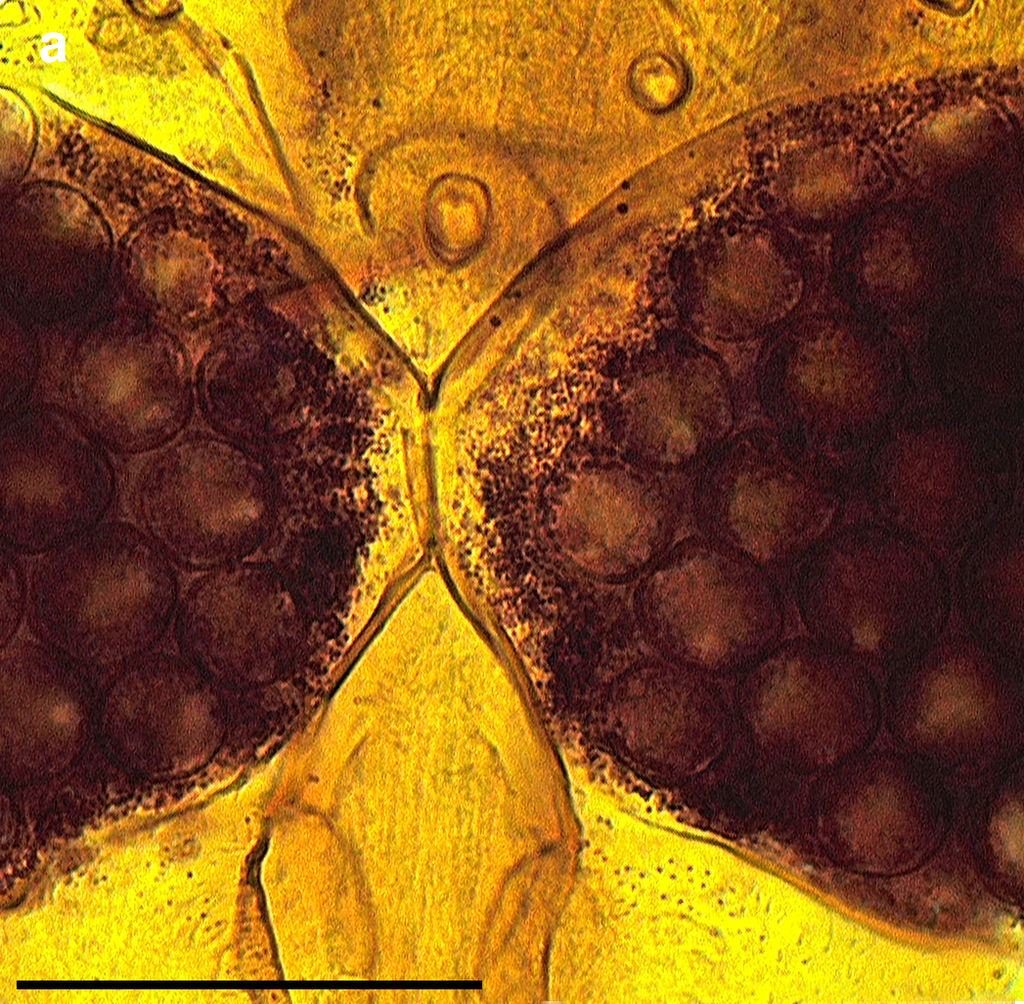
Vertex. Scale = 50 µm.

**Figure 3b.**
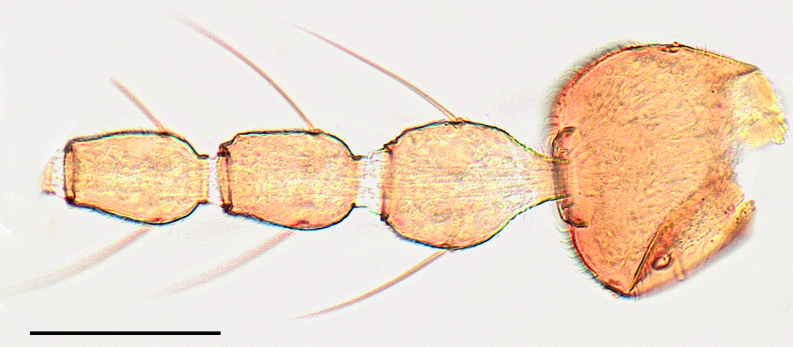
Antennal pedicellus and first three flagellomeres. Scale = 50 µm.

**Figure 3c.**
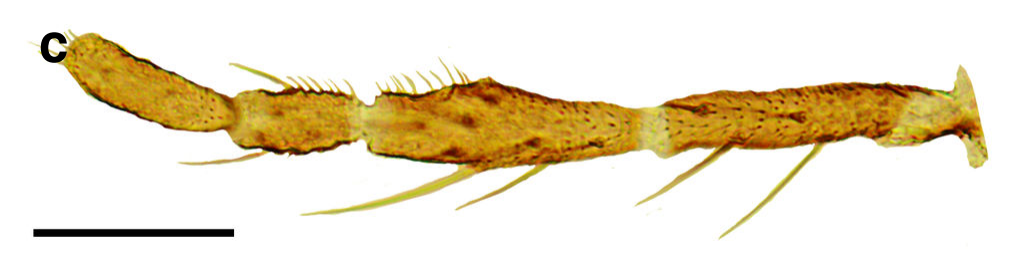
Left palp, dorsal view. Scale = 50 µm.

**Figure 3d.**
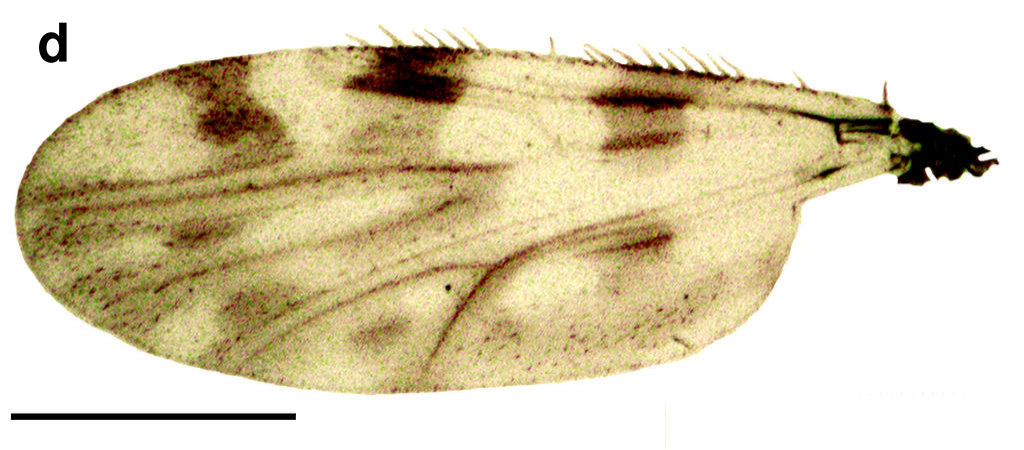
Wing, brightfield photo. Scale = 500 µm.

**Figure 3e.**
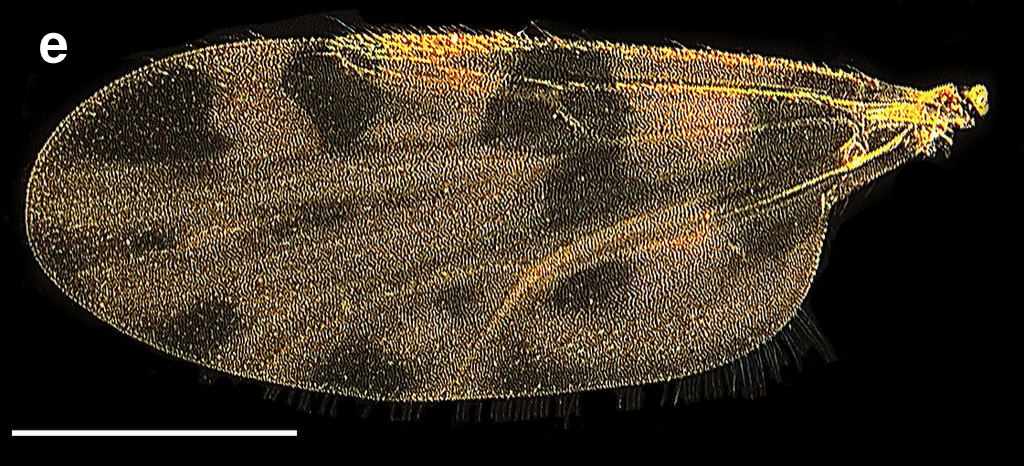
Wing, darkfield photo. Scale = 500 µm.

**Figure 3f.**
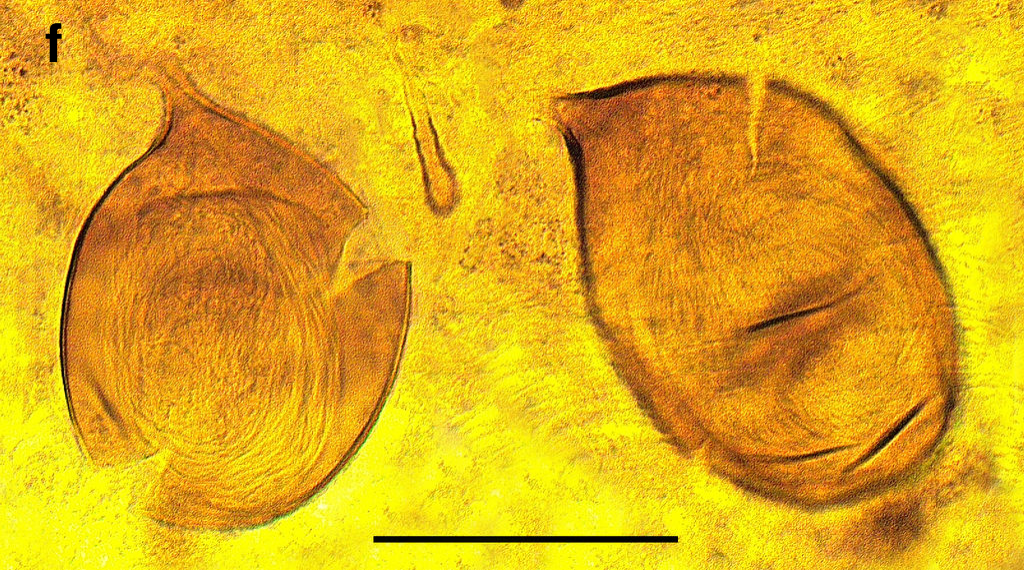
Spermathecae. Scale = 50 µm.

**Table 1.** Pairwise morphometric comparisons between three new species of *Culicoides (Culicoides)* and the morphologically most similar Scandinavian species, including divergence in COI sequences. The significance of differences between measurements was determined by multiple comparison test after Kruskal-Wallis (P_K-w_< 0.05) followed by a Conover-Inman test for all pairwise comparisons (Nielsen and Kristensen 2015). Comparisons that are significantly different are shown in yellow highlight. 1 - Species; 2 - Flagellum, length (μm); 3 - Antennal ratio (AR: length of flagellomeres 9–13 divided by length of flagellomeres 1–8); 4 - First flagellomere, length/width; 5 - Maxillary palp, length (μm); 6 - Maxillary palp ratio PR (length/width of third palp segment); 7 - Maxillary palp ratio P3/P2 (length of third maxillary palp segment divided by length of second); 8 - Length of wing (μm); 9 - Spermatheca ratio S/R; 10 - Head/proboscis ratio; 11 - Mandibular teeth; 12 - Maxillary teeth; 13 - Ratio M/M; 14 - Fronto-vertex /ocellus; 15 - Antennal sensilla coeloconica.

**1**	**2**	**3**	**4**	**5**	**6**	**7**	**8**	**9**	**10**	**11**	**12**	**13**	**14**	**15**
*C. pulicaris*	742± 26	1.09± 0,03	1.52± 0.07	255.6± 14.3	2.9± 0.2	0.84± 0.05	1626± 67	121± 0.08	1.19± 0.04	16.7± 1.2	19.5± 1.1	1.17± 0.08	1.2± 0.3	15.47± 1.97
*C. boyi*	746± 44	1.03± 0.04	1.78± 0.07	245.1± 17.7	2.9± 0.3	1.04± 0.11	1641± 10	1.05± 0.3	1.29± 0.07	15.1± 1.2	17.1± 1.4	1.13± 0.10	1.5± 0.2	14.71± 1.26
Divergence of COI sequences = 16.5%														
*C. newsteadi*	591± 45	1.04± 0.05	1.53± 0.15	193.6± 18.7	2.6± 0.2	1.11± 0.10	1291± 12	1.04± 0.02	1.32± 0.13	13.4± 1.4	15.9± 1.5	1.18± 0.20	0.3± 0.3	7.70± 0.67
*C. selandicus*	616± 10	1.12± 0.04	1.56± 0.11	216.8± 8.6	3.2± 0.3	0.96± 0.06	1339± 33	ND	1.16± 0.06	15.0± 1.0	19.6± 1.5	1.31± 0.12	1.5± 0.8	12.29± 0.95
Divergence of COI sequences = 16.2%														
*C. newsteadi*	591± 45	1.04± 0.05	1.53± 0.15	193.6± 18.7	2.6± 0.2	1.11± 0.10	1291± 12	1.04± 0.02	1.32± 0.13	13.4± 1.4	15.9± 1.5	1.18± 0.20	0.3± 0.3	7.70± 0.67
*C. kalix*	646± 14	1.13± 0.04	1.46± 0.06	212.4± 4.0	2.9± 0.2	0.87± 0.08	1423± 39	1.17± 0.08	1.29± 0.07	12.8± 0.6	14.9± 1.5	1.17± 0.12	1.2± 0.3	10.70± 0.82
Divergence of COI sequences = 15.6%														
*C. selandicus*	616± 10	1.12± 0.04	1.56± 0.11	216.8± 8.6	3.2± 0.3	0.96± 0.06	1339± 33	ND	1.16± 0.06	15.0± 1.0	19.6± 1.5	1.31± 0.12	1.5± 0.8	12.29± 0.95
*C. kalix*	646± 14	1.13± 0.04	1.46± 0.06	212.4± 4.0	2.9± 0.2	0.87± 0.08	1423± 39	1.17± 0.08	1.29± 0.07	12.8± 0.6	14.9± 1.5	1.17± 0.12	1.2± 0.3	10.70± 0.82
Divergence of COI sequences = 5.9%														
